# Echocardiographic parameters of severe pulmonary regurgitation after surgical repair of tetralogy of Fallot

**DOI:** 10.1111/chd.12762

**Published:** 2019-03-07

**Authors:** An Van Berendoncks, Roderick Van Grootel, Jackie McGhie, Matthijs van Kranenburg, Myrthe Menting, Judith A.A.E. Cuypers, Ad J.J.C. Bogers, Maarten Witsenburg, Jolien W. Roos‐Hesselink, Annemien E. van den Bosch

**Affiliations:** ^1^ Department of Cardiology Erasmus Medical Center Rotterdam The Netherlands; ^2^ Department of Cardiology Antwerp University Hospital Antwerp Belgium; ^3^ Department of Cardiothoracic Surgery, Erasmus Medical Center Rotterdam The Netherlands

**Keywords:** CMR, echocardiography, pulmonary regurgitation, TOF

## Abstract

**Aims:**

Reliable evaluation of the severity and consequences of pulmonary regurgitation (PR) in patients with repaired tetralogy of Fallot (TOF) is crucial to timely identify the need for pulmonary valve intervention. We aimed to identify the accuracy of echocardiographic parameters to differentiate between moderate and severe PR, using phase contrast cardiac magnetic resonance imaging (CMR) as gold standard.

**Methods and results:**

In this cross‐sectional study, 45 TOF patients with both echocardiographic and CMR measurements of PR were enrolled. All quantitative and semiquantitative echocardiographic measurements such as pressure half time (PHT), Color flow jet width (CFJW), ratio CFJW/right ventricle outflow tract (RVOT) diameter, PR index and the presence of early termination of the PR jet, end‐diastolic antegrade flow and diastolic backflow in main pulmonary artery (MPA), and PA branches correlated significantly with PR fraction on CMR. Qualitative assessment with color flow on echocardiography overestimated PR Multivariate linear regression analysis identified the ratio of CFJW/RVOT diameter and PHT as independent predictors of PR fraction. Accuracy of echo parameters was tested to differentiate between mild‐to‐moderate and severe PR Combining different echocardiographic parameters increased sensitivity and specificity. The addition of diastolic flow reversal in the PA branches to PHT below 167 milliseconds increased the NPV from 87% to 89% and PPV from 62% to 76%.

**Conclusions:**

Comparison with CMR confirms that echocardiographic parameters are reliable in predicting PR severity. Combined measurement of diastolic flow reversal in the pulmonary artery branches and PHT is reliable in the detection of severe PR in the follow‐up of TOF patients.

## 1 INTRODUCTION

Pulmonary regurgitation (PR) is common after surgical repair of tetralogy of Fallot (TOF) and pulmonary valve replacement (PVR) is advised before the onset of irreversible right ventricular dysfunction.^1^, ^2^ The optimal timing of PVR, especially in the absence of symptoms or signs of hemodynamic or electrical instability, remains subject of debate. The desired restoration of RV size and function must be put against the risk of multiple interventions over the course of a patient’s lifetime, keeping in mind that the long‐term outcome of PVR has yet to be shown.^1^ However, with the introduction of percutaneous treatment options, such as placing a “Melody‐valve,” a lower threshold for intervention might arise. Determining the severity of PR and evaluating the consequences of this PR on the right ventricle (RV) is crucial in the follow‐up of patients with TOF.

Phase contrast cardiac magnetic resonance imaging (CMR) is considered the gold standard for the assessment of PR severity, RV dilatation, and function.^3^, ^4^, ^5^ However, since CMR is time‐consuming, expensive, and not accessible to patients with claustrophobia and/or implanted cardiac devices, echocardiography is considered the first‐line screening modality for the assessment of PR in routine daily practice.^6^, ^7^ Although echocardiography is widely available and used, interpretation of PR is largely qualitative^1^ or semiquantitative and contradictory results have been published regarding the predictive value and accuracy of these measurements compared to CMR.^5^, ^8^, ^9^, ^10^, ^11^, ^12^, ^13^, ^14^, ^15^, ^16^, ^17^ In our experience, not all cardiologists are familiar with the different possibilities and opportunities of echocardiography, and knowledge on adequate PR assessment is lacking. Furthermore, the optimal threshold of the different measurements is not uniformly defined.^14^, ^18^ Finally, Doppler signals can be influenced by a restrictive RV physiology, mimicking severe PR in the presence of only mild pulmonary insufficiency.

The aim of this study is (1) to describe all echocardiographic parameters of PR and evaluate their accuracy to differentiate between mild‐to‐moderate and severe PR compared with the gold standard CMR and (2) to identify the (combination of the) most simple, accurate, and ready to use echocardiographic parameter(s) for reliable estimation of PR in the follow‐up of operated TOF patients in daily clinical practice.

## 2 METHODS

### 2.1 Study population

All consecutive patients who underwent surgical repair of TOF in the Erasmus MC, Rotterdam, The Netherlands between 1968 and 2000 at age <15 years and who participated in the “quality‐of‐life” study,^19^ and had echocardiography and CMR were recruited for this cross‐sectional study. The methods and results of this long‐term longitudinal follow‐up study have been previously described.^19^ The in‐hospital cardiac examination included medical history, physical examination, standard 12‐lead ECG, echocardiography, and CMR. All investigations were aimed to be performed on the same day. In the current study, we have focused on the CMR and echocardiographic findings at last follow‐up. Patients were excluded from this substudy if no CMR imaging was performed (due to ICD/pacemaker, claustrophobia, rejected, or no show). The institutional Medical Ethical Committee approved the study (METC nr 2010‐015). Written informed consent was obtained from all patients.

### 2.2 Echocardiography

A comprehensive 2‐dimensional transthoracic echocardiogram in harmonic imaging was performed using an iE33 ultrasound system (Philips Medical Systems, Best, The Netherlands) equipped with a transthoracic broad‐band S5‐1 or a broad‐band X5‐1 matrix transducer.

Chamber measurements, including left ventricular ejection fraction (EF) (Simpson’s method), RV fractional area change (FAC), and tricuspid annular plane systolic excursion (TAPSE) were performed conform guidelines of the American Society of Echocardiography.^20^ Valvular regurgitation and stenosis were evaluated according to the European Association of Echocardiography recommendations.^16^, ^17^

**Figure 1:**
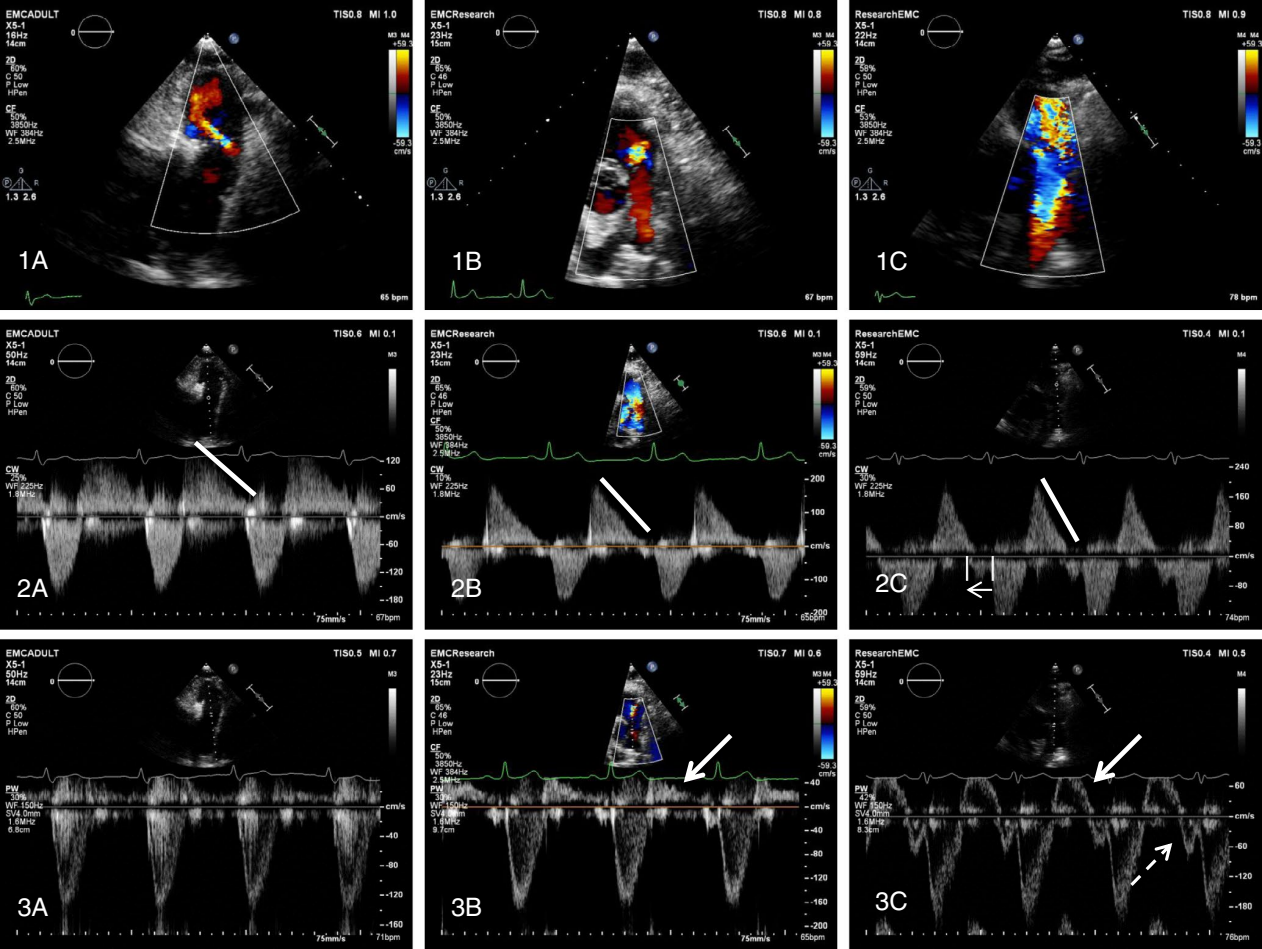
Differentiation of PR severity using color flow, CW, and PW Doppler echocardiography. Assessment of PR severity seen in the parasternal short‐axis view in a patient with mild (left), moderate (center), and severe (right) PR. Top: Qualitative assessment of PR severity using color‐flow imaging. 1A, Small regurgitant jet, no diastolic flow reversal in the main pulmonary artery (MPA) or PA branches. 1B, Increased width of the color flow jet and presence of diastolic flow reversal in the MPA. 1C, A broad color flow jet covering the total diameter of the RVOT and diastolic flow reversal coming from the right PA branch. Mid: Continuous Doppler recording of PR with PHT (white line). 2A, Slow flow deceleration of the PR jet during the entire diastole. 2B, more rapid flow deceleration during the entire diastole. 2C, rapid flow deceleration during diastole with early termination of the PR jet indicated by the small arrow. Bottom: pulsed‐wave Doppler in the MPA. 3A No signs of diastolic flow reversal in the MPA. 3B Mild diastolic flow reversal in the MPA (dense white arrow). 3C, Diastolic flow reversal in the MPA (dense white arrow) with end diastolic antegrade forward flow (dashed arrow)

In addition, the following parameters were assessed for PR severity (Figure 1):

- Recorded by color flow Doppler in the parasternal short‐axis view:
  ○ Proximal color flow jet width (CFJW) of the PR,
  ○ Ratio of the CFJW to the right ventricle outflow tract (RVOT) diameter,
  ○ Detection of diastolic flow reversal in the main pulmonary artery (MPA) and the left or right pulmonary branch (LPA, RPA).
- Evaluated using continuous‐wave Doppler:
  ○ Deceleration time,
  ○ Pressure half time (PHT) measurement,
  ○ Duration of the PR in diastole and the PR index (=100* PR/diastole duration ratio)
- Evaluated with both continuous wave Doppler and pulsed‐wave Doppler at RVOT level and at the level of the tips of the pulmonary valve:
  ○ the presence of early termination of PR
  ○ the presence of end‐diastole antegrade flow (Figure 1).

### 2.3 CMR imaging

CMR imaging was performed with a Sigma 1.5‐T whole‐body scanner (GE Medical Systems, Milwaukee, Wisconsin) with dedicated phased‐array cardiac surface coils. Details of the MR sequence used have been reported previously.^19^ For CMR analyses, a commercially available Advanced Windows workstation (GE Medical Systems) was used, equipped with Q‐mass (version 5.2, Medis Medical Imaging Systems, Leiden, The Netherlands). The ventricular volumetric data set was quantitatively analyzed by a single investigator (JAAEC) using manual outlining of endocardial borders in end systole and end diastole, excluding large trabeculae (visible on three subsequent slices) and the papillary muscles from the blood volume. Biventricular end‐diastolic volume, end‐systolic volume, EF, and valvular regurgitation fractions were calculated and compared with reference values.

### 2.4 Statistical analysis

Continuous data are presented as mean ± standard deviation (SD) or median with interquartile ranges when appropriate. Categorical variables are presented as frequencies and percentages. To quantify correlations, we used the Pearson correlation test for continuous variables or Spearman rank correlation test when appropriate. Multivariate linear regression analysis according to the stepwise method was performed for associations between echocardiographic parameters and PR fraction calculated with CMR. The following 5 parameters: ratio CFJW/annulus, PHT, time pulmonary insufficiency in diastole, antegrade flow end‐diastolic, and retrograde flow in PA branches (variables with a *P* value of less than .1 in univariate analysis) were added to the multivariable model. The multivariable model was tested for collinearity (variance inflation factor [VIF] score). A linear regression of PR fraction from CMR with the ratio CFJW/RVOT diameter and CW Doppler PHT to identify clinically meaningful cutoff points at, respectively, 20 and 40% PR measured on CMR. Patients were categorized according to the regurgitation fraction (RF) calculated on CMR into 3 categories: mild (<20%), moderate (20%‐40%), and severe (>40%). The kappa coefficient was calculated to assess the agreement between visual assessment of PR and PR measured with CMR. Proportions in mild to moderate versus severe PR were compared using Fisher’s exact test. Sensitivity, false‐positive rate (1‐specificity), positive predictive value (PPV), and negative predictive value (NPV) of echocardiographic parameters to grade PR using the RF found on CMR (dichotomized at 40%) as the gold standard were calculated for each echocardiographic parameter.

An interobserver reproducibility assessment was performed on 20 randomly selected patients for the RVOT diameter, CFJW, ratio CFJW/RVOT, and PHT using the Bland‐Altman method. The coefficient of variability was calculated as the standard deviation of the difference of the two measurements divided by the mean of the two measurements, and multiplied by 100%. *P* values of ≤.05 (2‐sided tests) were considered to be statistically significant. Statistical analysis was performed using the statistical packages IBM SPSS Statistics version 21.0 (IBM SPSS Statistics, IBM Corporation, Armonk, New York).

## 3 RESULTS

### 3.1 Study population

The baseline characteristics of the 45 patients with repaired TOF included in this study are summarized in Table 1. The mean age was 33 ± 9 years, with a range from 18 to 51 years and 18 patients underwent PVR after initial corrective surgery, 1 had a percutaneous (Melody) valve replacement. The mean time between echo and CMR was 1.5 months. Mean PR fraction measured with CMR was 31 ± 21% and considered as mild PR in 16 (35%), moderate PR in 13 (29%), and severe PR in 16 (35%) patients.

**Table 1:** Baseline characteristics and echocardiographic parameters of patient population (*n* = 45)

Variable	Total patient population (*n* = 45)	Mild‐to‐moderate PR (*n* = 29)	Severe PR (*n* = 16)
*Patient characteristics*			
Age (years)	33 ± 9	34 ± 9	33 ± 8
Gender (% male)^a^	(23) 60	(18) 67	(5) 45
Ethnicity (% Caucasian)^a^	100	100	100
BMI^a^	24.7 ± 4.8	24.8 ± 4.5	24.6 ± 5.7
NYHA^a^	93% NYHA I, 7% NYHA II	93% NYHA I, 7% NYHA II	93% NYHA I, 7% NYHA II
*Surgical details*			
Age at surgical repair (year)^a^	3.4 ± 3.0	3.4 ± 3.0	3.4 ± 3.0
Time since surgical repair (years)^a^	29 ± 8	30 ± 8	28 ± 8
*Type of surgical repair* *a*			
No patch (%)	4 (9)	4 (17)	0
Transannular patch (%)	31 (69)	18 (75)	13 (100)
RVOT patch (%)	2 (4)	2 (8)	0
*Echocardiography*			
*PR assessment (eyeball)* *b*			
None (%)	2 (4)	2 (8)	0
Mild (%)	8 (18)	8 (33)	0
Moderate (%)	5 (11)	5 (21)	0
Severe (%)	24 (53)	9 (37)	15 (100)
PHT (ms)	174 (139)	227 (91)	130 (47)
PHT <100 ms	4 (9)	0 (0)	4 (25)
Color flow jet width (ms)	19 (18)	13 (9)	27 (5)
Ratio CFJW/RVOT diameter	0.97 (0.63)	0.57 (0.34)	0.98 (0.05)
PR index	0.80 (0.32)	0.87 (0.15)	0.72 (0.12)
Early termination of PR jet (%)	30 (67)	15 (52)	15 (94)
End‐diastolic antegrade flow (%)	24 (53)	11 (39)	13 (81)
Diastolic flow reversal in MPA (%)	33 (73)	17 (58)	16 (100)
Diastolic flow reversal in PA branches (%)	24 (53)	8 (28)	16 (100)
PV peak systolic velocity (m/s)*	2.2 ± 0.8	2.4 ± 0.8	2.0 ± 0.7
TAPSE (mm)	20 ± 5	20 ± 5	19 ± 5
RVFAC (%)*	41 ± 10	43 ± 11	38 ± 8

Abbreviations: BMI, body mass index; CFJW, color flow jet width; MPA, main pulmonary artery; NYHA, New York Heart Association class; PA, pulmonary artery; PHT, pressure half time; PR, pulmonary regurgitation; PV, pulmonary valve; RVFAC, right ventricular fractional area change; RVOT, right ventricular outflow tract; TAPSE, tricuspid annular plane systolic excursion.

Variables are expressed as number (%), mean (±SD) or median (interquartile range).

^a^ Data available for n = 37 patients.

^b^ Data available for 39 patients.

Mean peak systolic velocity of the pulmonary valve of the total population was 2.2 ± 0.8 m/s including mild PV stenosis in 15 (33%), a moderate PV stenosis in 7 (16%), and a severe PV stenosis in 1 (2%) patient. Right ventricular (RV) function (available for 39 patients) was found normal in 15 patients (33%), mildly impaired in 18 patients (40%), and moderately impaired in 6 (13%) patients. Quantitative RV evaluation demonstrated a reduced RVFAC <35% in 8 patients indicating preserved RV function in the majority of patients (Table 1).

### 3.2 Evaluation of the accuracy of echocardiographic PR analysis

Qualitative assessment of PR on echocardiography indicated the presence of severe PR in more than half of the patients and showed moderate agreement with the 3 PR categories (mild PR <20%, moderate 20 >  PR <40%, severe PR >40%) according to PR fraction measurement on CMR (kappa coefficient of agreement 0.465, *P* < .01) (Table 2). Qualitative PR assessment on echocardiography correctly identified all patients with severe PR; however, PR severity was overestimated in a substantial number of patients (25%) (Table 2). Echocardiographic qualitative and quantitative measurements of PR severity are listed in Table 1.

**Table 2:** Agreement between echocardiographic assessment and CMR measurement of PR

Qualitative assessment of PR on echocardiography	CMR PR fraction			Total
	Mild PR <20%	Moderate 20 < PR <40%	Severe PR >40%	
None‐mild	10	0	0	10
Moderate	2	3	0	5
Severe	1	8	15	24
Total	13	11	15	39

Abbreviations: CMR, cardiac magnetic resonance; PR, pulmonary regurgitation.

Qualitative assessment of PR on echocardiography was available for 39 out of the 45 patients.

**Figure 2:**
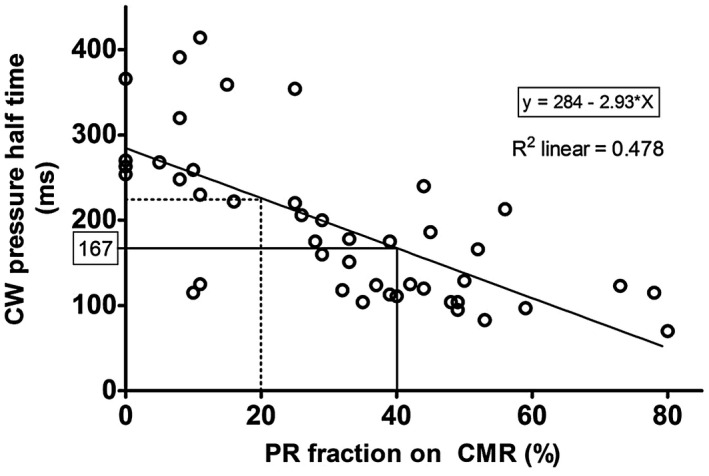
Scatter plot of echocardiographic pressure half time and PR fraction on CMR. Regression equation: *y* = 2,84E2+ −2.93*x*; *r *= −0.691; *P* < .0001

Univariate and multivariate regression analysis of the different echocardiographic parameters affecting PR are listed in Table 3. Both the ratio CFJW/RVOT diameter and PHT were significantly associated with the PR fraction on CMR. The cutoff points for, respectively, ratio CFJW/RVOT diameter and PHT at 20% and 40% PR were derived from linear regression analysis (Figure 2).

**Table 3:** Univariate and multivariate linear regression analysis

	Univariate			Multivariate		
	Beta	*R*	*P* value	Beta	*R*	*P* value
Diameter PV annulus (mm)^a^	0.587	0.345	<.001			
Color flow jet width (mm)^a^	0.816	0.665	<.001			
Ratio CFJW/RVOT diameter	0.737	0.543	<.001	0.491	0.610	.002
Pressure half time (ms)	−0.691	0.478	<.001	−0.334		.028
Deceleration time (ms)^a^	−0.691	0.478	<.001			
Early termination PR jet^a^	0.609	0.371	<.001			
Time diastole (ms)^a^	−0.238	0.056	.116			
Time PI in diastole (ms)	−0.540	0.292	<.001			
End‐diastolic antegrade flow	0.506	0.256	<.001			
Retrograde flow main PA^a^	0.608	0.370	<.001			
Retrograde flow in PA branches	0.648	0.420	<.001			

Total model including the following five parameters: ratio CFJW/annulus, PHT, time PI in diastole, antegrade flow end‐diastolic and retrograde flow in PA branches explained 61% of the pulmonary regurgitation fraction measured with CMR. Collinearity statistics revealed no significant collinearity between these 5 parameters (range VIF score 1.5‐4). Stepwise multivariate regression analysis indicated that both the ratio CFJW/annulus and PHT remained significantly associated with PR fraction on CMR.

Abbreviations: CFJW, color flow jet width; PA, pulmonary artery; PI, pulmonary insufficiency; PR, pulmonary regurgitation; PV, pulmonary valve; RVOT, right ventricular outflow tract.

^a^ Parameters not included in the multivariate model due to collinearity and degrees of freedom.

Accuracy of echocardiographic parameters was tested to differentiate between mild‐to‐moderate and severe PR (dichotomized at 40%). An overview of the differentiate capacity and the predictive values of all echocardiographic parameters are listed in Table 4.

**Table 4:** Sensitivity and specificity of echocardiographic variables in differentiating between mild to moderate (0%‐40%) and severe (>40%) PR on CMR

Variable	*P* value	Sensitivity	Specificity	PPV	NPV
	Fisher’s exact test				
Early termination of PR jet	.07	94	48	50	93
End‐diastolic antegrade flow	.011	81	61	54	85
Diastolic flow reversal in MPA	.003	100	41	48	100
Diastolic flow reversal in LPA branch	<.001	100	71	67	100
Ratio CFJW/RVOT diameter >0.77	<.001	100	64	61	100
PHT <100 ms	.013	25	100	100	70
PHT <167 ms	<.001	81	71	62	87
Ratio CFJW/RVOT diameter >0.77 and PHT <100 ms	.012	25	100	100	70
Ratio CFJW/RVOT diameter >0.77 and PHT <167 ms	<.001	81	82	72	88
Diastolic flow reversal LPA and PHT <167 ms	<.001	81	86	76	89
Diastolic flow reversal LPA and early termination of PI jet	<.001	93	72	65	95

Abbreviations: CFJW, color flow jet width; LPA, left pulmonary artery; MPA, main pulmonary artery; PHT, pressure half time; PI, pulmonary insufficiency; PR, pulmonary regurgitation; RVOT, right ventricular outflow tract.

The presence of diastolic flow reversal in the main PA and in the PA branches, had excellent sensitivity and NPV to identify severe PR However, specificity values are rather low. PHT on the contrary shows excellent specificity of 100% and a PPV of 100%. Based on the linear regression analysis, the optimal cutoff value to detect severe PR at 40% in our study population is a PHT of less than 167 milliseconds (Figure 2). Using this cutoff value, the sensitivity for detection of severe PR significantly increased to 81% with both good PPV and NPV (Table 4).

The ratio of the CFJW over RVOT diameter showed good correlation with PR fraction (Table 3). To test the accuracy of this parameter, the optimal cutoff point for the ratio of the CFJW to RVOT diameter was calculated. Using the cutoff of 0.77, the sensitivity of the ratio CFJW to RVOT diameter was 100% to detect a severe PR To improve the specificity, we combined both PHT with a cutoff of 167 milliseconds and CFJW ratio with a cutoff at 0.77 (Table 4). However, the measurement of both CFJW and RVOT diameter may be difficult to correctly delineate on the echocardiogram. An interobserver reproducibility assessment indicated a coefficient of variation (COV) of 1.29% for both RVOT diameter as for PHT. However, a very large variation (COV 29%) was present for CFJW measurements, resulting in a 25% COV in the CFJW/RVOT measurements. We, therefore, sought for easier applicable echocardiographic parameters. The combined presence of diastolic flow reversal in the PA branches and early termination of the PR jet or in combination with low PHT was found to be as accurate as the combination of PHT and CFJW ratio/RVOT, but much easier and just as reliable to detect.

**Figure 3:**
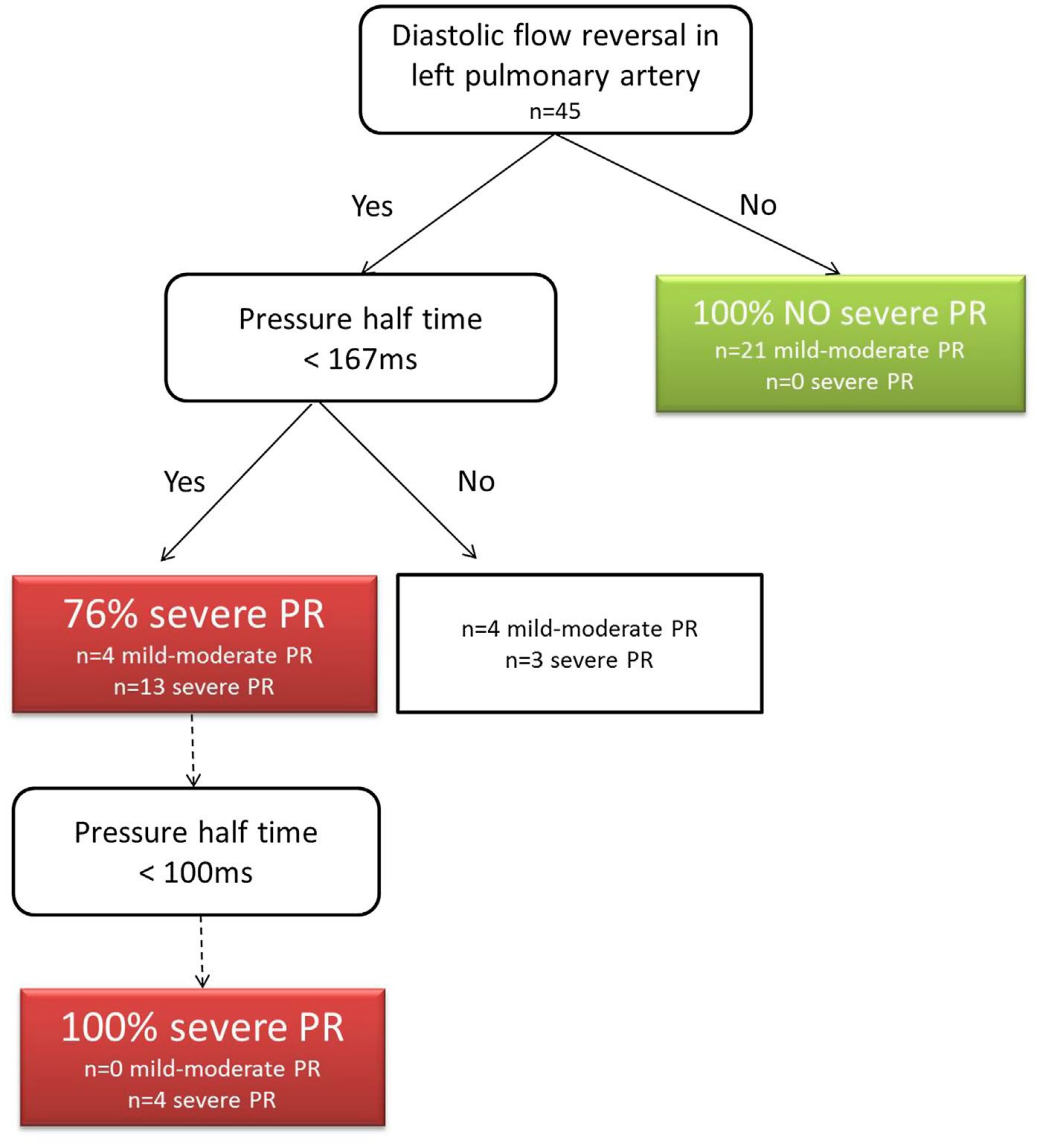
Suggestion for clinical decision tree. Flowchart to visualize decision rule for severe PR based on specificity and sensitivity analysis described in Table 4. Percentages represent probabilities that severe PR (>40% PR on CMR) will be detected by echocardiography using the combination of diastolic flow reversal in pulmonary artery branches and pressure half time

Figure 3 shows the clinical decision tree based on specificity and sensitivity analysis. The first split is made based on the question whether or not diastolic flow reversal in the pulmonary artery (PA) branches is present. The second step, is whether or not the PHT is below 167 milliseconds. In agreement with the negative predictive value of 100%, the probability of severe PR in the absence of diastolic flow reversal in the PA branches is 0%. If pulmonary flow reversal in the PA branches is present and the PHT is below 167 milliseconds, the probability of detecting a severe PR increased to 76%, even to 100% in case of PHT <100 milliseconds.

## 4 DISCUSSION

This study aimed to evaluate the accuracy of 10 different echocardiographic parameters in the assessment of PR severity in the operated adult TOF population. First, PR is highly prevalent with moderate to severe PR being present in 67% of patients. Second, qualitative assessment of PR on echocardiography is good, but overestimates moderate PR Third, all 10 investigated quantitative and semiquantitative echocardiographic measurements are accurate to detect severe PR Fourth, combining different echocardiographic parameters increased sensitivity and specificity. As such, the combination of diastolic flow reversal in the PA branches and short PHT is suggested as the best, ready to use and reliable measurement to identify severe PR in TOF patients.

Different cutoff points of PR fraction on CMR have been used to quantify PR with a PR fraction of >20% considered as hemodynamically significant PR^8^, ^14^ and PR fraction above 40% indicating severe PR^5^, ^9^, ^12^ In line with the previous studies, we used the CMR cutoff values of <20% for mild PR, 20%‐40% to indicate moderate PR and >40% for severe PR As the assessment between mild and severe PR is easy to differentiate qualitatively with color flow jet, we choose to test the accuracy of the echocardiographic parameters to differentiate between mild‐to‐moderate and severe PR This differentiation is clinically relevant and an important determinant for good follow‐up of TOF patients.^21^

Our study demonstrates that the presence of backflow from the PA branches is an excellent and accurate parameter to identify severe PR Flow reversal from the PA branches, indicating a larger volume of flow from further downstream, is not only intuitively but also statistically more accurate in detecting severe PR compared with flow reversal in the MPA. These findings are in line with flow reversal from the descending aorta in severe aortic regurgitation and have been demonstrated previously.^9^

PHT appeared to be a good predictor of severe PR, both as a continuous and dichotomous variable.^8^, ^9^, ^14^ The more severe the PR, the more rapid equalization of right ventricular and PA pressure occurs and the shorter the PHT. A specificity of 100% can be explained by the fact that only 4 patients in our study had a PHT value of less than 100 milliseconds and all were classified as severe PR Based on the linear regression analysis a cutoff value of 167 milliseconds is suggested, this clearly improved the sensitivity in detecting severe PR in our study population. Caution, however, is needed as PHT can also be influenced by diastolic properties of the right ventricle.^21^ Elevated right ventricular diastolic properties or restrictive right ventricular function can be found in postoperative patients, which may induce likewise early and rapid equilibration of diastolic pressures resulting in early termination of PR jet, reduction of PR index and the presence of end‐diastolic antegrade flow.^22^ Evaluation of PR severity solely based on Doppler analysis of the RVOT with early termination of the PR jet and end‐diastolic antegrade flow, could suggest the presence of severe PR Indeed, our study demonstrates that the presence of early termination of the PR jet and the presence of antegrade flow are individually well correlated with PR severity. However, if color flow mapping does not indicate flow reversal in the MPA or only shows a small PR jet width, the previous finding is compatible with mild PR and restrictive right ventricle physiology. Information of additional parameters is needed to exclude false‐positive results in the presence of restrictive PR physiology. In our study, this is well illustrated with the combined presence of backflow in the PA branches and the presence of end‐diastolic antegrade flow. Early termination of the PR jet or a short PHT improves this accuracy to detect severe PR Moreover, the combined presence of backflow in the PA branches and the presence of a PHT <167 milliseconds is as accurate as the more complex measurements of a ratio CFJW/RVOT diameter >0.77 and a PHT <167 milliseconds, a combination of parameters that recently has been demonstrated to be highly accurate to identify significant PR in a larger TOF population.^14^ Both the identification of backflow in the PA branches as the presence of a short PHT represent clear cut and easy to measure parameters, essential for a reliable evaluation of PR severity in patients with repaired TOF.

## 5 CLINICAL RECOMMENDATIONS

Echocardiography is recommended as first‐line diagnostic method for initial and longitudinal assessment of PR in TOF patients.^6^, ^7^ Grading of PR severity, however, remains difficult as standards for PR quantification are less robust than those for aortic regurgitation.^16^, ^17^ Recently combined measurements PHT, slope, jet‐to‐RVOT ratio have been suggested in the follow‐up of TOF patients.^9^, ^14^, ^15^ Measuring CFJW is, however, affected by a large interobserver variation. We investigated all available echocardiographic parameters for PR severity and found that the combination of backflow in the PA branches and a short PHT is as accurate as the more complex measurements. We, therefore, recommend this ready to use and easier approach to assess PR severity in TOF patients:

1. Evaluation with color flow over PV, PA, and PA branches from the parasternal short‐axis view at the level of the aortic valve.
2. If no backflow from the PA branches is present, we can exclude severe PR.
3. If diastolic flow reversal from the PA branches is present, we can further differentiate between moderate and severe PR using continuous wave Doppler analysis. If both backflow in PA branches is visualized and PHT is short, severe PR is present.

Careful follow‐up should be organized in patients with severe PR However, the prognostic value and implications on clinical follow‐up (timing of referral for PVR) and frequency of follow‐up visits need to be evaluated in a prospective follow‐up study and larger patient population.

## 6 LIMITATIONS

The following limitations need to be acknowledged. Firstly, the sample size is limited and secondly, this is a cross‐sectional study which implies that clinical implications of the described findings need to be evaluated in a large, prospective follow‐up study. Thirdly, the median interval between the echocardiogram and CMR exam was 1.5 months. The study population included 2 outliers with, respectively, 353 and 368 days between both exams. Both patients, however, had an echocardiogram one year before and one year after the CMR. As the severity of the PR on echocardiogram had not changed through these 2 years (PHT was, respectively, 220 milliseconds and 223 milliseconds for the first patient and 153 milliseconds and 158 milliseconds for the second patient) the patients remained in the study cohort. Although no significant clinical changes were noted during the interval, it is possible that the severity of PR may have varied in relation to their hemodynamic state.

## 7 CONCLUSIONS

This study demonstrates that echocardiography is accurate in predicting PR severity in the follow‐up of TOF patients. In particular, straightforward measures including the presence of diastolic flow reversal from the PA branches and a PHT <167 milliseconds are able to differentiate mild‐to‐moderate from severe PR Although each of the investigated parameters showed excellent correlation with the CMR PR fraction, a combination of different parameters further improved the accuracy and differentiates from restrictive RV physiology. The calculated cutoff value of PHT as well as the suggested flow chart requires further validation in a larger study population prior to the clinical application.

## CONFLICTS OF INTEREST

The authors declare that they have no conflict of interest with the contents of this article.

## AUTHOR CONTRIBUTIONS

All authors had substantial contributions to the research design, data acquisition, analysis or interpretation, revised the manuscript, and gave their approval for submission of the finale version.

*Concept/design*: An Van Berendoncks, Jackie McGhie, Jolien W Roos‐Hesselink; Annemien E van den Bosch

*Data analysis/interpretation*: An Van Berendoncks, Roderick Van Grootel, Jackie McGhie, Annemien E van den Bosch Drafting article, An Van Berendoncks

*Critical revision of article*: Roderick Van Grootel; Jackie McGhie, Judith A A E Cuypers; Ad J J C Bogers, Maarten Witsenburg, Jolien W Roos‐Hesselink, Annemien E van den Bosch

*Statistics*: An Van Berendoncks; Roderick Van Grootel

*Data collection*: Jackie McGhie; Matthijs van Kranenburg, Myrthe Menting; Judith A A E Cuypers.
